# Muscle weakness has a limited effect on motor control of gait in Duchenne muscular dystrophy

**DOI:** 10.1371/journal.pone.0238445

**Published:** 2020-09-02

**Authors:** Ines Vandekerckhove, Nathalie De Beukelaer, Marleen Van den Hauwe, Benjamin R. Shuman, Katherine M. Steele, Anja Van Campenhout, Nathalie Goemans, Kaat Desloovere, Marije Goudriaan

**Affiliations:** 1 Department of Rehabilitation Sciences, KU Leuven, Leuven, Belgium; 2 Clinical Motion Analysis Laboratory, University Hospitals Leuven, Pellenberg, Belgium; 3 Department of Child Neurology, University Hospitals, Leuven, Belgium; 4 Mechanical Engineering, University of Washington, Seattle, Washington, United States of America; 5 WRF Institute for Neuroengineering, University of Washington, Seattle, Washington, United States of America; 6 Department of Development and Regeneration, KU Leuven, Leuven, Belgium; 7 Department of Orthopedics, University Hospitals Leuven, Leuven, Belgium; 8 Department of Human Movement Sciences, VU University, Amsterdam, the Netherlands; University of Pittsburgh, UNITED STATES

## Abstract

**Aim:**

Our aim was to determine if synergy weights and activations are altered in Duchenne muscular dystrophy (DMD) and if these alterations could be linked to muscle weakness.

**Methods:**

In 22 children with DMD and 22 typical developing (TD) children of a similar age, surface electromyography (sEMG) of the gluteus medius, rectus femoris (REF), medial hamstrings, tibialis anterior, and medial gastrocnemius (GAS) were recorded during gait. Muscle weakness was assessed with maximal voluntary isometric contractions (MVIC). Synergies were calculated with non-negative matrix factorization. The number of synergies explaining ≥90% of the variance in the sEMG signals (N90), were extracted and grouped with k-means cluster analysis. We verified differences in weights with a Mann-Whitney U test. Statistical non-parametric mapping (Hotelling's T^2^ test and two-tailed t-test) was used to assess group differences in synergy activations. We used Spearman’s rank correlation coefficients and canonical correlation analysis to assess if weakness was related to modifications in weights and activations, respectively.

**Results:**

For both groups, average N90 was three. In synergy one, characterized by activity at the beginning of stance, the DMDs showed an increased REF weight (p = 0.001) and decreased GAS weight (p = 0.007). Synergy activations were similar, with only a small difference detected in mid-swing in the combined activations (p<0.001). Weakness was not associated with these differences.

**Conclusion:**

Despite the apparent weakness in DMD, synergy weights and activations were similar between the two groups. Our findings are in line with previous research suggesting non-neural alterations have limited influence on muscle synergies.

## Introduction

Duchenne muscular dystrophy (DMD) is an X-linked degenerative muscular disorder and is the most common of the muscular dystrophies with a prevalence of one in 3500–6000 male births [1–3]. In individuals with DMD, the protein dystrophin is absent. This protein is expected to play an important role in ensuring the stability of the muscle cell membrane and protecting the muscles from contraction induced damage [4–6]. When the disease progresses, muscle damage will get the overhand and muscles will get infiltrated with non-contractile fibrofatty tissue [3]. This is the cause of the most prominent symptom in DMD, i.e. muscle weakness [7].

Muscle weakness can be the result of altered motor commands due to 1) lesions in the central nervous system (CNS) [8], 2) disruptions in signal transmission between the CNS and the muscles [9], and/or 3) changes in the muscle itself [3,7]. In children with DMD, the main cause for muscle weakness appears to be the changes in muscle structure [3]. There is no evidence to support that muscle weakness in DMD has a neurological cause [9,10].

Muscle weakness is expected to play an important role in the deterioration of walking ability in children with DMD. However, no direct association between lower limb muscle weakness and altered gait kinematics and kinetics has been found [11]. Even though muscle weakness in DMD has no neural components, it could be considered a constraint, which the CNS needs to account for when initiating or controlling a task [12]. Therefore, it can be expected that muscle weakness will have an influence on motor control of gait. By analyzing if and how muscle weakness affects motor control of walking, we might better understand the association between muscle weakness and decreased walking ability in children with DMD.

Synergy analysis is often used to assess motor control of gait [13–21]. Muscle synergies are defined as “consistent patterns of multi-muscle coordination that generate a specific action” [22]. From synergy analysis, the total variance accounted (tVAF) for by a specified number of synergies can be used to quantify complexity of motor control, with higher values indicating reduced complexity of motor control [19,23]. Additionally, synergy weights and activations can be analyzed. Synergy weights are the contributions of each muscle in a synergy and synergy activations represent the timing and the amount of activation of each synergy [19]. Synergies are expected to be flexible, thereby able to react to internal and external biomechanical constraints without affecting the intendent movement [24,25]. Therefore, as muscle weakness is an internal biomechanical constraint, muscle synergies during gait might be altered in children with DMD [12].

However, Goudriaan et al. (2018) determined that synergy complexity, represented by the total variance accounted for by one synergy (tVAF_1_) was not different between DMD and typical developing (TD) children, even though the children with DMD were significantly weaker [15]. In the same study, a higher tVAF_1_ was seen in children with a brain lesion (i.e. cerebral palsy) of a similar age and with an equal level of muscle weakness as the children with DMD. Based on these findings, the authors hypothesized that synergy complexity could be considered a quantification of the neural capacity of an individual and that biomechanical constraints, such as muscle weakness, only have a limited effect on complexity of motor control of gait.

Although muscle weakness did not change tVAF_1_, it might have an effect on synergy weights and activations. DMD is characterized by muscle-specific alterations [7]. It can be expected that muscle weakness of a specific muscle group will be visible in synergy weights, because they represent the individual contribution of a muscle to a synergy. Consequently, activations patterns might also change. There is evidence that muscle activity of DMD gait is indeed altered when compared to TD children [2,26]. For example, Melkonian et al. (1980) were the first to report “out of phase” muscle activity of the tibialis anterior (TIA) and posterior, peroneus brevis and longus, soleus (SOL) and gastrocnemius in children with DMD [27]. In addition, Sutherland et al. (1981) and Ropars et al. (2016) showed increased activity of the rectus femoris (REF) and TIA showed increased activity, while gluteus medius (GLU) activity was decreased [2,26]. Both studies attributed these changes in muscle activity to a compensation mechanism for lower limb muscle weakness and an attempt to increase in joint stability during gait, since the brain areas responsible for the motor control are intact in children with DMD [28]. However, the association between altered muscle activity and lower limb muscle weakness has never been confirmed. Synergy analysis provides a tool to assess the effect of muscle weakness on not only one muscle group or an agonist-antagonist couple, but on multiple, simultaneously activated muscles (e.g. synergies). By getting a better understanding of how muscle weakness affects motor control of gait in children with DMD, current clinical care focusing on prolonging walking ability can be further specified. In DMD this is considered important in order to maintain a certain level of functionality, as well as to postpone spinal deformities and muscle contractures [2,3,29].

Therefore, the goal of this study was to determine if muscle weakness has an influence on motor control of gait in children with DMD. We used synergy analysis to assess motor control of gait in a group of children with DMD (5–17 years) and TD children of a similar age. We extracted synergy weights and activations and compared them between the two groups (DMD and TD). We then determined if these potential differences could be attributed to muscle weakness in four lower limb muscle groups: knee extensors (KE), knee flexors (KF), dorsiflexors (DF), and plantar flexors (PF). Based on previous research [2,26], we hypothesized that motor control of gait is altered in DMD and that this would be expressed in both altered synergy weights and activations. Since muscle activity was shown to be related to the Vignos functional score in children with DMD [26], we expected that altered weights and activations would be correlated with lower limb muscle weakness.

## Materials and methods

### Participants

Three-dimensional gait analyses (3DGA) of 22 children with DMD and 22 TD children were assessed for this research. Data of 15 children with DMD and 14 TD children with a similar age were from a previous published study [15]. This retrospective dataset was amended with seven additional children with DMD extracted from the database of neuromuscular reference center in the University Hospital of Leuven. We used the following inclusion criteria: (1) diagnosed with DMD via immunohistochemistry, muscle biopsy and/or mutation of the dystrophin gene, (2) no previous lower-limb surgery, (3) between five and 18 years old, (4) able to walk independently for at least 100 meters, and (5) corticosteroid treatment and participation in clinical trials were allowed. Eight additional TD children of a similar age as the children with DMD were recruited via colleagues and students from the Clinical Motion Analysis Laboratory (CMAL) of the University Hospitals of Leuven (Pellenberg) and included in the study if they had no neurological or neuromuscular disorder. All children, except one TD child, performed additional strength measurements by means of dynamometry.

This study was approved under the Declaration of Helsinki by the local ethics committee (Commissie Medische Ethiek KU Leuven; under S56041, S60321 and S61324). After agreeing to participate in this study, participants of 12 years of age or older and the participants’ parents or caretakers signed a written informed consent. All children were measured at the CMAL.

### Data collection

Similar to the previous study [15], a standard clinical exam (range of motion and strength by manual muscle testing) and 3DGA at self-selected speed were performed. Markers were attached according to the lower limb plug-in-gait model and marker trajectories were recorded with a 10–15 camera system (Vicon- UK Oxford, UK). Ground reaction forces were collected with two force plates embedded in the walk way (AMTI, Watertown, MA, USA). Gait cycles were manually defined in Nexus (Vicon Nexus 2.5). Muscle activity of the GLU, REF, vastus lateralis (VAL), medial hamstrings (MEH), biceps femoris (BIF), medial gastrocnemius (GAS), SOL and TIA was measured bilaterally with a 16-channel telemetric surface electromyography (sEMG) system (Zerowire, Cometa, Italy) at 1000 or 1500 Hz. The sEMG electrodes were attached according to the Seniam recommendations [30]. Muscle weakness of KE, KF, DF and PF was assessed according to Goudriaan et al. (2018a). The KE, KF, DF and PF maximal voluntary contractions (MVIC) were thereby collected in a standardized test position, that ensured a reduction of influence of assessor strength and compensation mechanisms. Depending on the child’s cooperation and level of fatigue, unilateral or bilateral MVICs were collected. In case of an unilateral assessment, the MVICs were only performed on the weakest side, which was determined based on strength outcomes of the clinical exam. In case no weakest side could be determined, the evaluated side was defined in a random way (by flipping a coin).

### Data analysis

Based on the strength outcomes of the clinical exam, the weakest side of the DMD and TD children was determined. Only sEMG and MVICs of this side were used in further analyses, except for one TD child of whom the data of the stronger side was included since the quality of the sEMG on the weaker side was poor.

Ten representative gait cycles containing good quality sEMG of the GLU, REF, MEH, TIA and GAS were selected. The sEMG signals were filtered with a 6^th^ order Butterworth bandpass filter with cut-off frequencies of 20 and 450 Hz. After rectifying, the sEMG signals were smoothed with 4^th^ order Butterworth lowpass filter with a frequency of 10 Hz [15,31]. We resampled the filtered sEMG signals of each step to 101 data points, representing 0–100% of a gait cycle. The resampled, filtered sEMG signals per individual step were normalized to the average amplitude of that step per child, for each muscle [15,32].

We used non-negative matrix factorization (NNMF) to calculate muscle synergies from the sEMG signals for each step [15,33,34]. The settings of the NNMF function in MATLAB (The Mathworks Inc., Natick, M.A., 2017a) were: 50 replicates, 1000 max iterations, 1*10^−4^ minimum threshold for convergence, and a 1*10^−6^ threshold for completion [15,34]. NNMF decomposes the sEMG signals into two matrices, i.e. W and C Matrix. W contains the synergy weights and C the synergy activations [19] such that:

$$sEMG=(W_{m*n}*C_{n*t})+error$$ (1)

where n is the number of synergies (one to four), t is the number of data points (101), m is the number of muscles (five), and error is the difference between the measured sEMG and the reconstructed sEMG. With the error value, tVAF_n_ was calculated as:

$${tVAF}_{n}=(1-\frac{\left[\sum_{j}^{t}\sum_{i}^{m}{\left(error\right)}^{2}\right]}{\left[\sum_{j}^{t}\sum_{i}^{m}{\left(EMG\right)}^{2}\right]})$$ (2)

The average tVAF_n_ of 10 steps for each child was determined. The number of synergies that could explain more than 90% of the variance in the sEMG signals (N90) were extracted and averaged for both groups (DMD and TD). Synergies weights were scaled in such a way that one represented the maximal weight of a muscle to a synergy. Then, the number of synergies for average value of N90 were grouped with k-means cluster analysis and the average synergy weights and activations of 10 steps for each child was calculated.The maximal force (N) from the MVIC of three representative trials for each muscle group was averaged and net joint torques normalized to bodyweight (Nm·kg^-1^) were calculated by multiplying the average maximal force with the moment arm (which was standardized at 75% of the segment length) and dividing by the body weight of the child [35].

Higher levels of subcutaneous fibrofatty tissue as well as in the muscles could act as an additional lowpass filter [36–38], thereby potentially influencing the outcomes of the synergy analysis [31]. Therefore, we also assessed the frequency distribution of the sEMGs signals with the PWELCH function in MATLAB. We used a window size of 1024 samples, an overlap op 512 samples, and 500 points for the Fourier transform. Next, we calculated the median frequency curves as well the absolute median frequency per muscle for both groups (TD and DMD).

### Statistical analysis

Normality of the MVICs, tVAF_1_ and synergy weights was checked with the Shapiro-Wilk test. Since we planned to use statistical (non) parametric mapping (S(n)PM) to assess differences in synergy activations between DMD and TD, normality of the synergy activations was assessed with a built-in function in SPM (SPM1d version 0.4, available for download at http://www.spm1d.org/). S(n)PM accounts for the covariance between related signals [39,40]. Therefore, as the synergistic patterns may demonstrate simultaneous activity during some intervals of the gait cycle, S(n)PM could be useful for determining alterations in the combined activity of muscle synergies.

Since the data could not be proven normally distributed, non-parametric statistics were used to address each research question.

With a Mann-Whitney U (MWU) test we evaluated whether the children with DMD were weaker than the TD children and if there was a difference in tVAF_1_ between the two groups. We also used the MWU-test to compare synergy weights between the two groups. We used the z-score from the MWU-test divided by the square root of the sample size to calculate the effect sizes (r) (Eq 3) [41].

$$r=\frac{z}{\surd (N)}$$ (3)

Synergy activations between DMD and TD were compared with a two-sample non-parametric Hotelling’s *T*^2^ (SnPM{*T*^2^}) [39,40]. If significant differences between the two groups were found with SnPM{*T*^2^}, we performed a non-parametric post-hoc two-tailed, two-sample t-test (SnPM{*t*}) to assess potential differences in the individual synergy activations [39,40].

In case of significant differences in synergy weights, we assessed whether the difference was associated with muscle weakness with Spearman’s rank correlation coefficients. The Altman classification (<0.20 = poor; 0.21–0.40 = fair; 0.41–0.60 = moderate; 0.61–0.80 = good and 0.81–1.00 = very good) was used to interpret the results [42]. The association between muscle weakness and potential changes in synergy activations was determined with a non-parametric canonical correlation analysis (SnPM{*X*^2^}).

To correct for multiple comparisons the Sidak threshold with an α-level of 0.05 was used and resulted in a corrected α-level of 0.01 for all analyses [43]. For the SnPM, the analyses number of iterations was set at 10000. In SnPM, small identified clusters are often unstable (e.g. they are not found when the analysis is run a second time). Therefore, we only considered clusters similar to or exceeding 5% of the gait cycle. All statistical analyses were performed in MATLAB (The Mathworks Inc., Natick, M.A., 2017a).

Finally, we used descriptive statistics to compare the outcomes of the frequency analysis.

## Results

In total, 22 boys with DMD (median age (interquartile range): 9.2 (3.5) years) and 22 TD children (8.7 (2.9) years) participated in this study (Table 1).

**Table 1:** Subject information of the children with DMD and TD children.

	DMD	TD
**Gender**	boys: 22	boys: 15; girls: 7
**Age [years]**	9.2 (6.8–10.3)	8.7 (7.8–10.7)
**Weight [kg]**	26.5 (19.9–35.0)	29.8 (25.0–37.1)
**Height [m]**	1.22 (1.11–1.31)	1.33 (1.29–1.46)

Abbreviations in alphabetic order: DMD = Duchenne muscular dystrophy; TD = typically developing.

**Fig 1:**
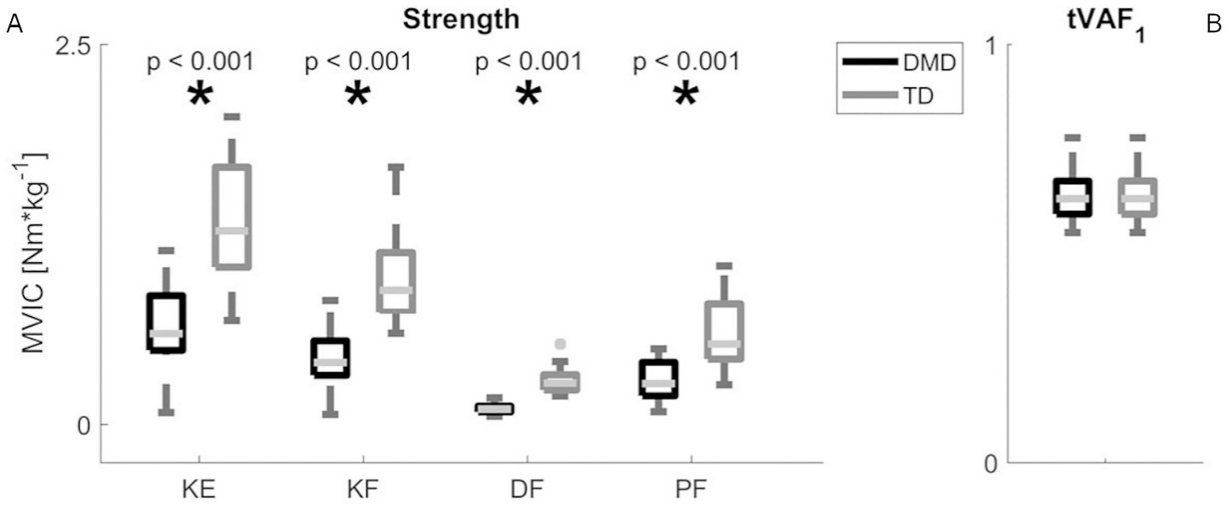
Results of MWU tests for MVICs and tVAF_1_ (α = 0.01). (A-B) show the median + interquartile ranges of the MVICs (A) of the KE, KF, DF and PF and tVAF_1_ (B). Significant differences between TD (grey) and DMD (black) based on the outcomes of MWU are indicated with an asterisk. Abbreviations in alphabetic order: DF = dorsiflexion; DMD = Duchenne muscular dystrophy; KE = knee extension; KF = knee flexion; MVIC = maximal voluntary isometric contraction; MWU = Mann-Whitney U; Nm·kg^-1^ = Newton meters per kilogram body weight; PF = plantar flexion; TD = typically developing; tVAF_1_ = total variance accounted for by one synergy.

The children with DMD (n = 22) were significantly weaker compared to the TD children (n = 21) in all muscle groups (p<0.001). No significant difference in tVAF_1_ between the DMD (n = 22) and TD children (n = 22) was found (p = 0.16). Fig 1 and Table 2 present a visual presentation and a more detailed overview of the MWU tests for MVICs and tVAF_1_, respectively.

**Table 2:** Group differences in MVICs and tVAF_1_ between DMD and TD from the MWU-test (α = 0.01) and their r effect sizes. Values are given in medians and 25^th^ and 75^th^ centiles.

	DMD	TD	MWU-test	Effect size
**KE MVIC [Nm·kg^-1^]**	0.60 (0.49–0.85)	1.27 (1.03–1.69)	**p<0.001**	**0.76**
**KF MVIC[Nm·kg^-1^]**	0.41 (0.32–0.55)	0.88 (0.74–1.13)	**p<0.001**	**0.79**
**DF MVIC [Nm·kg^-1^]**	0.10 (0.08–0.12)	0.27 (0.23–0.33)	**p<0.001**	**0.85**
**PF MVIC[Nm·kg^-1^]**	0.27 (0.19–0.41)	0.53 (0.43–0.79)	**p<0.001**	**0.65**
**tVAF_1_**	0.63 (0.59–0.67)	0.65 (0.64–0.69)	p = 0.16	0.21

Abbreviations in alphabetic order: DF = dorsiflexion; DMD = Duchenne muscular dystrophy; KE = knee extension; KF = knee flexion; MVIC = maximal voluntary isometric contraction; MWU = Mann-Whitney U; Nm·kg^-1^ = Newton meters per kilogram body weight; PF = plantar flexion; TD = typically developing; tVAF_1_ = total variance accounted for by one synergy.

**Fig 2:**
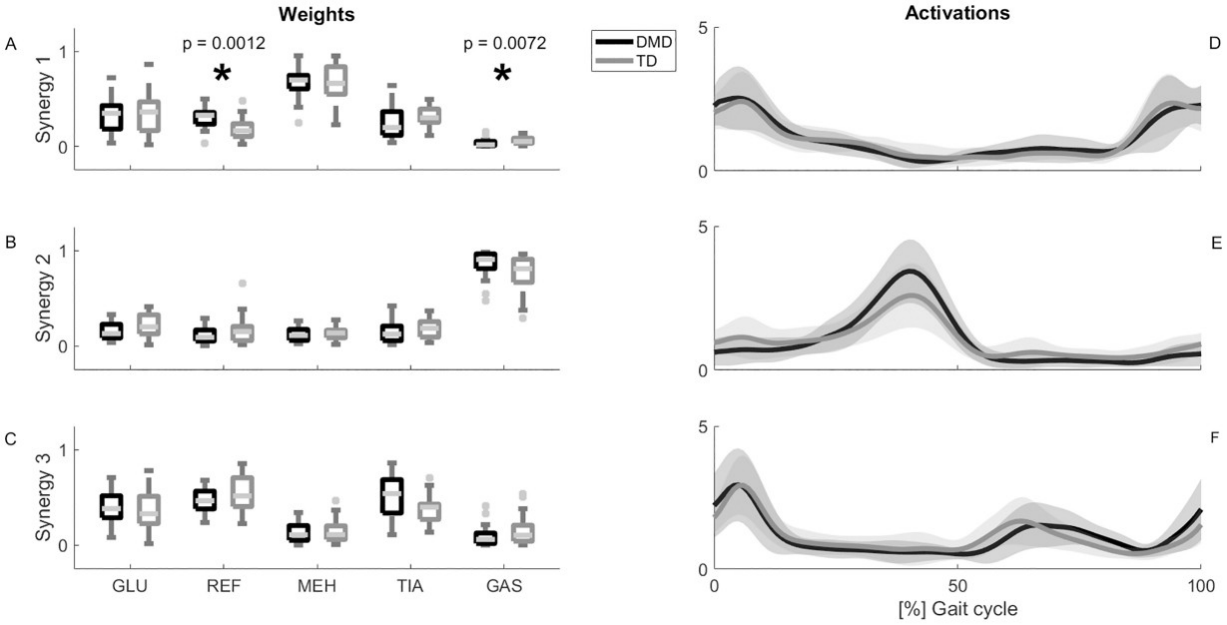
Results of SnPM {t} tests for the synergy activation curves and MWU tests for synergy weights (α = 0.01). Panel A-C show the median + interquartile ranges of the weight of the GLU, REF, MEH, TIA and GAS to synergy one (A), two (B) and three (C) respectively. Significant differences between TD (grey) and DMD (black) based on the outcomes of MWU are indicated with an asterisk. Panel D–F show the mean values + standard deviations of the activations of synergy one (D), two (E) and three (F) respectively. Significant differences between TD (grey) and DMD (black) based on the outcomes of SnPM {t} are indicated as black bars. Abbreviations in alphabetic order: DMD = Duchenne muscular dystrophy; GAS = gastrocnemius; GLU = gluteus medius; MEH = medial hamstrings; MWU = Mann-Whitney U; REF = rectus femoris; SnPM {t}, statistical non-parametric mapping post-hoc two-tailed two-sampled t-test; TD = typically developing; TIA = tibialis anterior.

The number of synergies was similar between groups, with an average N90 of 2.95 (0.21) for the children with DMD and 3 (0) for the TD children. Except for one DMD child (N90 = 2), N90 was three for all children. Therefore, we grouped three synergies with k-means cluster analysis. Synergy one is characterized by activity of the GLU and MEH at the beginning of the stance phase and at the end of swing phase. Synergy two is dominated by activity of the GAS and is active in midstance. In synergy three, the prime-movers are the REF and TIA and they are mostly active at the end of the stance phase and in the swing phase (Fig 2).

When comparing the weights of the five muscles between DMD and TD for the three synergies, we only found significant differences in the weights of two non-prime movers of synergy one. The children with DMD showed increased REF activity (0.33 (0.12)) in comparison with TD children (0.17 (0.13), p = 0.001, r = -0.49). A lower weight of the GAS was found for DMD (0.02 (0.04)) when compared to TD (0.05 (0.05) (p = 0.007, r = 0.41)(Fig 2 and S1 Table).

The SnPM {*T*^2^} revealed a significant difference in synergy activations between DMD and TD during swing (76–83% of the gait cycle; p<0.001). However, in the post-hoc analyses (SnPM{*t*}), this difference could not be replicated in the individual synergies (Fig 2 and S2 Table).

Only poor associations between MVIC-outcomes and the synergy weights were found for all three synergies (Table 3).

**Table 3:** Correlation between outcomes of strength measurement and synergy weights determined with Spearman’s rank correlation coefficients (ρ) in DMD.

	GLU	REF	MEH	TIA	GAS
	**Synergy one**				
**KE MVIC [Nm·kg^-1^]**	-0.11	-0.26	0.08	0.03	-0.10
**KF MVIC [Nm·kg^-1^]**	0.08	-0.17	-0.24	0.03	0.10
**DF MVIC [Nm·kg^-1^]**	-0.32	-0.15	0.29	-0.14	0.23
**PF MVIC[Nm·kg^-1^]**	0.22	-0.11	-0.36	0.09	-0.25
	**Synergy two**				
**KE MVIC [Nm·kg^-1^]**	-0.30	-0.03	0.20	-0.16	0.06
**KF MVIC [Nm·kg^-1^]**	-0.17	0.07	0.12	0.22	-0.03
**DF MVIC [Nm·kg^-1^]**	0.17	-0.33	-0.40	0.11	0.19
**PF MVIC [Nm·kg^-1^]**	-0.04	0.02	0.35	0.18	-0.30
	**Synergy three**				
**KE MVIC [Nm·kg^-1^]**	0.20	0.10	-0.11	-0.22	0.06
**KF MVIC [Nm·kg^-1^]**	-0.05	0.08	0.19	-0.21	0.23
**DF MVIC [Nm·kg^-1^]**	0.08	0.23	-0.16	0.00	-0.07
**PF MVIC [Nm·kg^-1^]**	-0.13	0.24	0.06	-0.10	0.13

Moderate to good correlations are in bold.

Abbreviations in alphabetic order: DF = dorsiflexion; DMD = Duchenne muscular dystrophy; GAS = gastrocnemius; GLU = gluteus medius; KE = knee extension; KF = knee flexion; MEH = medial hamstrings; MVIC = maximal voluntary isometric contraction; Nm·kg^-1^ = Newton meters per kilogram body weight; PF = plantar flexion; REF = rectus femoris; TIA = tibialis anterior.

*p < 0.01

**p < 0.001

**Fig 3:**
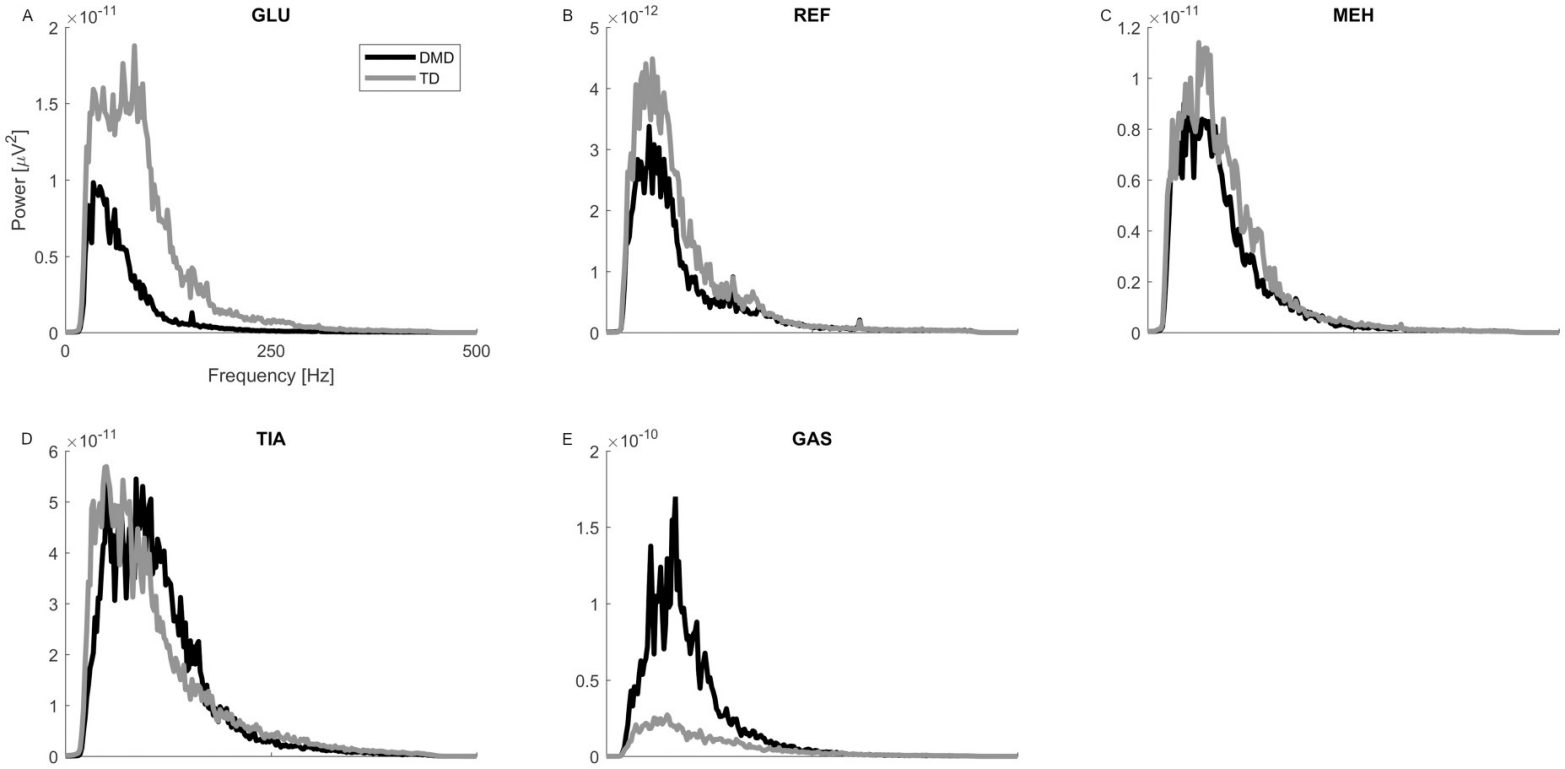
Power spectrum density plots of filtered sEMG signals (20–450 Hz). (A-E) show the median curves of the GLU (A), REF (B), MEH (C), TIA (D) and GAS (E) for the children with DMD (black) and the TD children (grey). Abbreviations in alphabetic order: DMD = Duchenne muscular dystrophy; GAS = gastrocnemius; GLU = gluteus medius; Hz = Hertz; MEH = medial hamstrings; REF = rectus femoris; TD = typically developing; TIA = tibialis anterior; μV = microvolts.

The frequency distribution curves can be found in Fig 3. The largest differences were found in the GLU and the GAS. The power spectrum density curve of the GLU was lower over the entire frequency band, while the curve of the GAS was higher in the children with DMD. Absolute median frequency values were lower in all muscles for the children with DMD: REF: 66.81, MEH: 83.39, TIA: 96.28, GAS: 82.89, GLU: 56.51 for the children with DMD. For the TD children these values were: REF: 73.97, MEH: 84.14, TIA: 102.86, GAS: 105.98, GLU: 82.35.

## Discussion

Muscle weakness is considered the main contributor to the pathological gait pattern in children with DMD. While no direct association between muscle weakness and altered kinematic and kinetic parameters has been found [11], we hypothesized that muscle weakness should be considered a biomechanical constraint affecting motor control of gait in children with DMD. We found significant differences in REF and GAS weights between the two groups in synergy one, but these differences were not associated with muscle weakness. Synergy activations were largely similar between groups. Therefore, our hypothesis could not be confirmed.

Since the REF is not one of the prime movers of synergy one, the increased REF weights suggest an increase in level of co-contraction during loading response and at the end of swing phase. This is in line with the findings of Ropars et al. (2016), who reported increased coactivation of the REF and MEH during gait in children with DMD of a similar age. The quadriceps is one of the first muscle groups that starts to deteriorate and is characterized by muscle atrophy [3,44]. This atrophy results in muscle weakness and the available motor units are not able to generate sufficient force during gait without changing their recruitment pattern. A compensation mechanism could have been to recruit larger/or more motor units, resulting in an increased muscle activity, as observed in synergy one. However, we expected that this increased activity would also be visible in the other two synergies, especially in synergy three where the REF is a prime mover, but this was not confirmed. Additionally, we would also have expected an increase in GLU and MEH weights to compensate for the decreased amount of contractile fibers, as these muscles are also atrophic and MEH demonstrated increased coactivation with the REF in a previous study [7,26,45]. Contrary, GLU and MEH weights were not different between the two groups (S1 Table).

Since in DMD, the type II fibers with the larger motor units are the first to deteriorate [46], sEMG amplitude would be lower than in TD children. However, to compensate, more motor units of the type I fibers could be recruited or the CNS can make use of temporal summation of the available motor units [47]. The net result would be an amplitude similar as in TD children. This could explain the similarity in GLU, REF, and MEH weights between the two groups (DMD and TD).

The calf muscles show a slightly different pattern. The muscle fibers are initially hypertrophic, albeit of poor quality [7]. Additionally, contrary to the quadriceps, the GAS is often prone to muscle contractures [48]. This increased stiffness could contribute to the net force production during gait [49], which would explain the decreased weight of the GAS in synergy one and the lack of a clear association between GAS weights.

Ropars et al (2016) found higher levels of co-contraction during walking in children with DMD [26]. This suggests that the agonist-antagonists couples were characterized by more and longer activity and/or by activity at different time periods during a gait cycle than in TD children. If this were the case, it would be visible in both synergy activation patterns as well as the weights of the individual muscles of the children with DMD. Contrarily, we also found similar synergy activation patterns in the children with DMD.

The higher levels of subcutaneous and intramuscular fibrofatty tissue could have reduced the amplitude of the sEMGs and acted as an additional lowpass filter [36–38]. In the sEMG this would have been visible as reduced variability and a lower power of the higher frequencies in the sEMGs (lower median frequency) [31,37]. The latter could be further increased by the early deterioration of the high frequency firing type II muscle fibers [46]. Indeed, we see a decrease in the median frequency in the children with DMD when compared to the TD children. However, the reduced variability would have been visible as a higher tVAF_1_ in the children with DMD. It appears that the changes in the frequency distribution have little effect on the outcomes of the synergy analysis. Nonetheless, it might be advisable to check and report the frequency distributions in participants who have the tendency towards increased and/or denser subcutaneous fat levels prior to further analyses.

While muscle weakness is expected to influence muscle activity on a smaller level (e.g. recruitment types and patterns), it appears to have a limited effect on the general activation patterns and muscle contributions during walking. There is evidence that part of muscle synergies are already present at a very young age [14]. With more walking experience, synergies are fine-tuned and expanded. Yet, in toddlers, muscle synergies are already very similar to the synergies of adults. Similarly, Shuman et al. (2019) determined that the composition of synergies are relatively fixed as treatments in children with cerebral palsy have only a small effect on the synergy weights [18]. The children in the study of Shuman et al. (2019) were approximately between four and 15 years old. The findings of these studies suggest that after a certain age, muscle synergies could be difficult to change. Since the age of our participants with DMD lies between 5 and 18 years old, their synergies might have been already developed. A longitudinal assessment of gait development in younger children with DMD, might provide more insight into how motor control of gait develops in this population, while adapting to progressive muscle weakness and other disease specific characteristics.

Even though the main effects of DMD are visible in the muscle, the brain-specific isoform of dystrophin is also absent. The lack of dystrophin in the brain has a negative effect on cognitive functions and has been associated with developmental disorders, including autism spectrum disorder [28]. However, no direct evidence that absence of the brain-specific isoform of dystrophin affects motor control of walking has been found [28]. Further, individuals with lesions in brain regions related to (gross) motor function, including walking, often show a higher tVAF_1_ [22]. This higher tVAF_1_ has been found in children with cerebral palsy, stroke survivors, and individuals with Parkinson’s disease, but not in children with DMD [15,22].

One of the limitations of our study, is our sample size might not have been sufficient to capture the heterogeneity of the children with DMD. The doses of corticosteroids, participation in clinical trials, use of wheel chair, performance on functional tests, i.e. six-minute walk test and north star assessment, differed among participants. Hence, disease progression is patient-specific, which has an effect on the generalizability of our results. To allow for a better comparison between different studies and to increase the generalizability of our results, we have added all relevant information of the children with the DMD in the supplementary materials available online (dx.doi.org/10.6084/m9.figshare.11288939), as well as the effect sizes of the MWU-tests (S1 Table and Table 2).

Even though walking is a complex bilateral motor task, we only measured sEMG of five muscles of the most affected side. In addition, we did not measure the weakness of the hip muscles, as our weakness assessment did not allow for a reliable measurement of those muscles. However, we do not expect large differences in synergies between DMD and TD with the use of a more expanded set of muscles, nor do we expect a significant relationship with hip muscle weakness. Since apparent weakness was already visible in the assessed muscles and almost no alterations and correlations were found.

Further, muscle synergies during gait, just like other kinematics and kinetics, are expected to be strongly related [50]. To tackle this problem of dependency within the dataset, two approaches have been commonly applied. First, the dependency can be taken into account through statistical methods. Mixed models have been used to correct for the correlation among multiple included gait cycles within the subjects [51]. Similarly, both sides for each subject can be included using mixed models, as this analysis takes into account the intra-subject variability (random effect) [52]. Secondly, many previous studies analyzed only one side (most commonly the most involved side) to ensure complete independency within the dataset. We selected the latter approach because this allowed us to reduce the assessment load and thereby control the impact of fatigue on the results. As only strength data of the weakest side was available, we included the unilateral sEMGs of the same side for synergy analysis.

The sEMG signals were not concatenated before running the NNMF, instead NNMF was run on the individual steps and the weights and activations were averaged afterwards. As a consequence, we did not account for the entire EMG variability present in the EMG step pattern. While this can affect the tVAF_1_ [53], Goudriaan et al (2018) who concatenated sEMG signals detected similar tVAF_1_ values [15]. Furthermore, we do not expect changes in the synergy weights and activations with concatenated sEMG signals, since we found the same synergy composition as previous studies that concatenated the sEMG signals [18].

We assessed muscle weakness via MVICs, which might not be representative of the muscle activity required during gait. For example, during gait, muscles vary in contraction types and do not act only isometrically. Additionally, motor control of MVICs was found to be different from motor control of gait [54]. This could explain the lack of stronger and more correlations between the MVIC outcomes and the changes in synergy weights. Despite these discrepancies, a muscle that produces a lower amount of force during a MVIC is also expected to produce a lower amount of force during concentric or eccentric contractions [55]. A possible explanation for the lack of a clear association between muscle weakness and gait pathology could be that the relationship is non-linear and might not have been detected with the statistical analyses applied in this and other studies [11,35,56]. The exploration of non-linear statistics could provide additional insight into the association between muscle weakness and gait pathology in future studies.

## Conclusion

Muscle weakness is one of the most important symptoms in DMD. Hence, it was expected that muscle weakness would be a constraint for motor control of gait. Although small modifications are visible, synergy weights and activations patterns appear to be similar between DMD and TD. Our findings are in line with previous research suggesting that non-neural alterations have limited influence on muscle synergies after a certain age. However, the recruited DMD sample was heterogenous, considering the corticosteroid doses, participation in clinical trials, the functional level etc. Hence, the results of the current study should be taken with some caution. Longitudinal analyses of walking, including the assessment of motor control via muscle synergy analysis, could provide more insight how the CNS copes with the increasing muscle weakness in children with DMD.

## Supporting information

S1 Table Group differences in synergy weights determined with the MWU-test (α = 0.01) and their r effect sizes. Values are given in medians and 25^th^ and 75^th^ centiles. Abbreviations in alphabetic order: DMD = Duchenne muscular dystrophy; GAS = gastrocnemius; GLU = gluteus medius; MEH = medial hamstrings; MWU = Mann-Whitney U; REF = rectus femoris; TD = typically developing; TIA = tibialis anterior.
(DOCX)

S2 Table All SnPM analyses (α = 0.01). Abbreviations in alphabetic order: DF = dorsiflexion; KE = knee extension; KF = knee flexion; MVIC = maximal voluntary isometric contraction; Nm·kg^-1^ = Newton meters per kilogram body weight; PF = plantar flexion; SnPM = statistical non-parametric mapping.
(DOCX)

S3 Table Correlation between age and synergy weights determined with Spearman’s rank correlation coefficients (ρ) in DMD. Moderate to good correlations are in bold. Abbreviations in alphabetic order: DMD = Duchenne muscular dystrophy; GAS = gastrocnemius; GLU = gluteus medius; MEH = medial hamstrings; PF = plantar flexion; REF = rectus femoris; TIA = tibialis anterior.
(DOCX)
