# Supplementary material for: Muscle weakness has a limited effect on motor control of gait in Duchenne muscular dystrophy
Source: PLoS One. 2020 Sep 2;15(9):e0238445. doi: 10.1371/journal.pone.0238445 (PMC7467330; doi:10.1371/journal.pone.0238445)
Supplement: S1 Table — Values are given in medians and 25th and 75th centiles. Abbreviations in alphabetic order: DMD = Duchenne muscular dystrophy; GAS = gastrocnemius; GLU = gluteus medius; MEH = medial hamstrings; MWU = Mann-Whitney U; REF = rectus femoris; TD = typically developing; TIA = tibialis anterior. (DOCX) [file pone.0238445.s001.docx]

**S1 Table.**

| Synergy weights | DMD | TD | MWU-test | Effect size |
| --- | --- | --- | --- | --- |
| Synergy one | | | | |
| GLU | 0.35 (0.18-0.43) | 0.36 (0.17-0.47) | p= 1.000 | 0.00 |
| REF | 0.33 (0.23-0.36) | 0.17 (0.11-0.24) | **p= 0.001** | **-0.49** |
| MEH | 0.67 (0.61-0.75) | 0.67 (0.55-0.84) | p= 0.897 | -0.02 |
| TIA | 0.20 (0.12-0.37) | 0.30 (0.25-0.39) | p= 0.016 | 0.36 |
| GAS | 0.02 (0.01-0.05) | 0.05 (0.03-0.09) | **p=0.007** | **0.41** |
| Synergy two | | | | |
| GLU | 0.13 (0.09-0.23) | 0.20 (0.13-0.33) | p= 0.093 | 0.25 |
| REF | 0.09 (0.05-0.17) | 0.15 (0.06-0.21) | p= 0.236 | 0.18 |
| MEH | 0.11 (0.06-0.18) | 0.14 (0.09-0.17) | p= 0.664 | 0.07 |
| TIA | 0.13 (0.06-0.21) | 0.19 (0.10-0.26) | p= 0.113 | 0.24 |
| GAS | 0.91 (0.81-0.97) | 0.81 (0.67-0.91) | p= 0.014 | -0.37 |
| Synergy three | | | | |
| GLU | 0.39 (0.29-0.52) | 0.33 (0.23-0.51) | p= 0.418 | -0.12 |
| REF | 0.47 (0.38-0.57) | 0.52 (0.41-0.71) | p= 0.256 | 0.17 |
| MEH | 0.11 (0.05-0.21) | 0.11 (0.06-0.20) | p= 0.953 | 0.01 |
| TIA | 0.54 (0.34-0.69) | 0.40 (0.27-0.43) | p= 0.056 | -0.29 |
| GAS | 0.06 (0.02-0.13) | 0.11 (0.04-0.21) | p= 0.142 | 0.22 |
