# Supplementary material for: Muscle weakness has a limited effect on motor control of gait in Duchenne muscular dystrophy
Source: PLoS One. 2020 Sep 2;15(9):e0238445. doi: 10.1371/journal.pone.0238445 (PMC7467330; doi:10.1371/journal.pone.0238445)
Supplement: S2 Table — Abbreviations in alphabetic order: DF = dorsiflexion; KE = knee extension; KF = knee flexion; MVIC = maximal voluntary isometric contraction; Nm·kg-1 = Newton meters per kilogram body weight; PF = plantar flexion; SnPM = statistical non-parametric mapping. (DOCX) [file pone.0238445.s002.docx]

**S2 Table.**

|  | Hotellings T^2^ test (SnPM{T^2^})  *Vector field analysis* | Post hoc two-sample t-test (SnPM{t}) | Canonical correlation (SnPM {χ^2^})  *Vector field analysis* |
| --- | --- | --- | --- |
| Synergy activations | T^2^* = 24.3732  Cluster1 = 75-83% GC  **p<0.001** | Synergy 1  t* = 4.1489  Clusters = 0  Synergy 2  t* = 3.9044  Clusters = 0  Synergy 3  t* = 4.0175  Cluster1 = 80% GC  **p = 0.005** | KE MVIC [Nm**^.^**kg^-1^]  χ^2^* = 18.5369  Clusters = 0  KF MVIC [Nm**^.^**kg^-1^]  χ^2^* = 18.7227  Clusters = 0  DF MVIC [Nm**^.^**kg^-1^]  χ^2^* = 19.1338  Clusters = 0  PF MVIC [Nm**^.^**kg^-1^]  χ^2^* = 18.6310  Clusters = 0  Age [years]  χ^2^* = 19.3792  Cluster1 = 3-5% GC  **p = 0.005**  Cluster2 = 46-47% GC  **p = 0.008** |
