# Supplementary material for: Muscle weakness has a limited effect on motor control of gait in Duchenne muscular dystrophy
Source: PLoS One. 2020 Sep 2;15(9):e0238445. doi: 10.1371/journal.pone.0238445 (PMC7467330; doi:10.1371/journal.pone.0238445)
Supplement: S3 Table — Moderate to good correlations are in bold. Abbreviations in alphabetic order: DMD = Duchenne muscular dystrophy; GAS = gastrocnemius; GLU = gluteus medius; MEH = medial hamstrings; PF = plantar flexion; REF = rectus femoris; TIA = tibialis anterior. (DOCX) [file pone.0238445.s003.docx]

**S3 Table.**

|  | GLU | REF | MEH | TIA | GAS |
| --- | --- | --- | --- | --- | --- |
|  | **Synergy one** | | | | |
| Age [years] | -0.07 | **0.55*** | 0.54 | **-0.68**** | 0.18 |
|  | **Synergy two** | | | | |
| Age [years] | -0.07 | -0.22 | **-0.59*** | -0.10 | **0.55*** |
|  | **Synergy three** | | | | |
| Age [years] | 0.12 | -0.31 | **-0.56*** | **0.71**** | -0.51 |

**p < 0.01*

***p < 0.001*
